# Species diversity of different insect families trapped under beer-based volatile fermentation

**DOI:** 10.1186/s13065-020-00699-x

**Published:** 2020-08-09

**Authors:** Mazher Farid Iqbal, Yu-Long Feng

**Affiliations:** 1 Liaoning Key Laboratory for Biological Invasions and Global Changes, College of Bioscience and Biotechnology, Shenyang Agricultural University, Shenyang, 110866 Liaoning Province People’s Republic of China; 2 Adaptive Research, Gujranwala, 52330 Punjab Province Pakistan

**Keywords:** Adult insect attraction, Bait traps, Bioactive compounds, Diversity indices, Gas Chromatography and Mass Spectrometry

## Abstract

**Background:**

Insect species composition is an important phenomenon playing a significant role in the ecosystem. Chemical control of insects and pests releases toxic materials to the environment. These chemicals are dangerous to human populations. In this situation, there is a dire need to develop strategies to overcome the haphazard use of chemicals. The present investigations were carried out to explore the diversity of different insects attracted through bait fermentation.

**Methods:**

The traditionally prepared bait fermentation was used to attract different insect populations both in treated (traps installed near field crops) and control traps (traps installed near invasive weed). Abundance, evenness, richness and equitability of these trapped insects were calculated. The chemical screening of bait fermentation was done using Gas Chromatography and Mass Spectrometry (GC–MS).

**Results:**

Significant difference (*P* < 0.05) in abundance of insect populations was found in treated compared to control trap. The insects of Noctuidae family recorded high Shannon- Wiener’s diversity index followed by Muscidae. Margalef’s index was recorded maximum in the treated traps (10.77) compared to those of control (8.09). The yielded index indicated that maximum richness was found in bait treated compared to control. The Shannon’s equitability’s values were investigated higher in Noctuidae (1.48), while, maximum evenness was observed in Muscidae (2.05) in treated trap. This fermentation was dried at room temperature and ground at 0.1 micron size. Our result showed significant (*P* < 0.05) effects of extraction times, with high yield in first extraction by polar solvents. *Co*-*efficient of determination* (*R*^*2*^= 0.87) recorded similar results in both extractions, however high root mean square error (0.97) recorded with bait + distilled water solvent showed linear arc line gave better performance. Finally, this fermentation was analyzed using GC–MS and recorded volatile compounds that were involved in the attraction of major and minor pests.

**Conclusion:**

Fermentation can help for the attraction of different families of insects of various crops. The field experiment suggested that this fermentation is economical, easily installed and consumed only 0.64 RMB/0.09 USD, including infrastructures per location. Bait fermentation is safe biochemical constituents and did not spread any toxic chemicals to the environment.
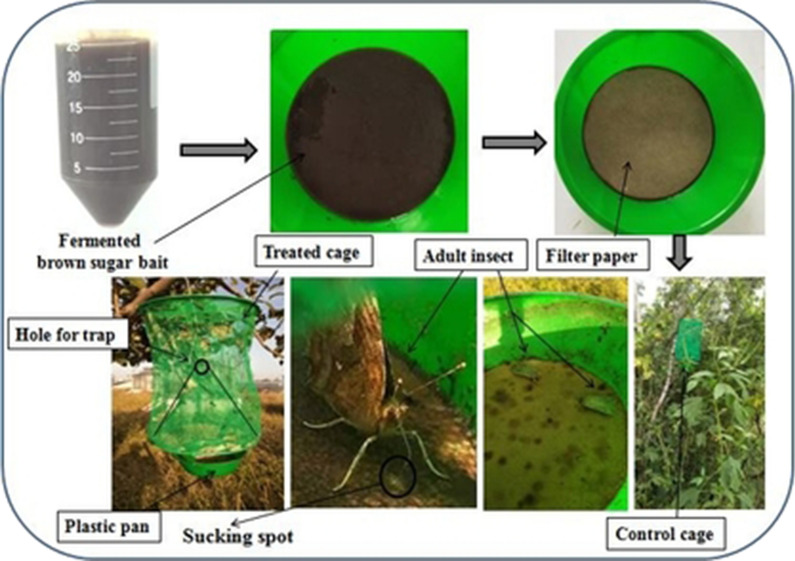

## Introduction

Synthetic pesticides are frequently used in agriculture to control different types of insects in the world. These pesticides are creating resistance against diversified insect-pests in maize, millet, soybean, sunflower, sesame and vegetable crops. These synthetic organic compounds spread toxic chemicals in the environment [1–4]. These toxic chemicals are causing respiratory diseases, skin itchiness, redness, and cardiovascular diseases, in human beings during the hand or aerial spray process [5–9]. These are also toxic to live stocks and birds because they are highly abundant dwellers of the field crops [10, 11]. In this situation, there is a dire need to develop suitable strategies to control insects of different field crops. Biological control agents, phytochemicals, pheromone traps, light traps and bait traps are helpful to manage this disaster [12–22]. Our present research played a vital role to attract the insects of different crops. Fermentation used to defeat this problem and acted like pheromone traps was made up of rotten fruits mixed with beer and brown sugar. The bait trap attracted the moths of different insect families [23–25]. Likewise, the primary sex pheromone was found in 1959 [26] and the insects of Lepidoptera were attracted by the sex pheromones [27]. In our experiment bait fermentation attracted both sex of various insects. Consequently, bait fermentations provide benefit over sex pheromones, because they can vwwb used for targeting a wide range of insects. Therefore, several trapping methods based on pheromones and kairomones are already in use for managing insects using different fermentations. So, food-based baits are an effective technique for insect control.

Firstly we hypothesized that abundance, richness and evenness of different insect families increased through using fermentation. Moreover, the yield would be great in the first extraction recorded after drying of each fermented solvents. Finally, GC–MS screening of bait fermentation may contain various volatile chemical constituents that can be involved in attraction of insect populations. How many insect families could be attracted by bait fermentation in both treatments and also how many chemicals could be screened out from fermentation through GC–MS technique? The current study was aimed to determine the abundance, richness, evenness, and equitability of the insect families attracted by fermentation. Moreover, the study also evaluated dried baits eluted with different solvents. However, *coefficient of determination* (*R*^*2*^) was calculated and compared to root mean square error (RMSE). Finally traditionally prepared sugar fermented fruit bait was analyzed using GC–MS after eluted by low and high polar solvents, and chemical activities of identified bioactive compounds were discussed with available literature.

## Methods

The present investigations were carried out to evaluate ecological indices such as abundance, evenness and species richness of different insects diversified in mountainous areas of Shenyang Agricultural University. The studied vicinity having 41.8282 ^o^N and 123.5647 ^o^E is the northeastern part in China edges South Korean borderline [28].

### Preparation of fermentation

The fermentation contained 500 g rotten fruits (banana, apple and peach taken in same quantity), was ground in 1 L distilled water (pH 7.3) and mixed in blender until homogenized [29–31]. This material was put in a 5 L plastic bottle, in which 330 mL 4% beer was added. One kilogram of brown sugar was mixed in this solution and stirred gently. The contents were preserved at room temperature (27 °C), stirred regularly, after 10 days the fermentation was ready for use.

### Installation of traps

**Fig. 1:**
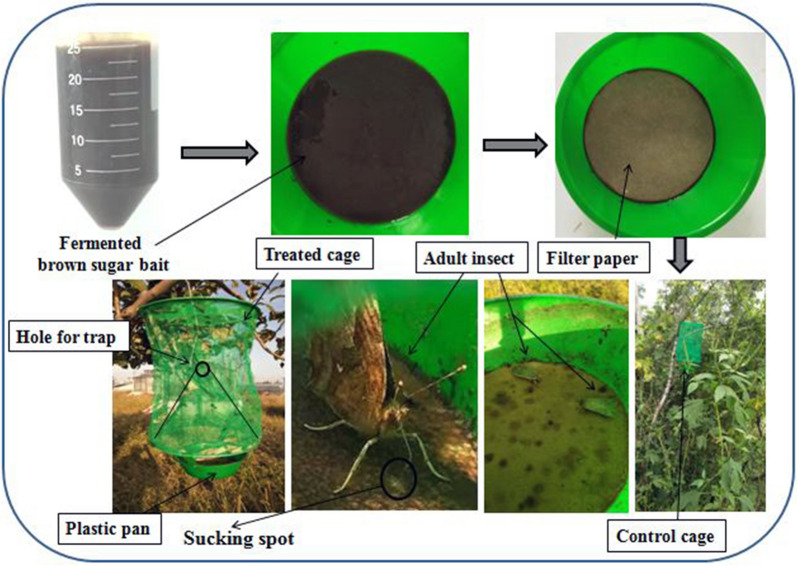
Graphical installation of bait fermentation traps

Paired traps were installed near the cropped area (treatment) along with non cropped area (control) at forth week of August by transect walk method in three different locations and repeated with three times. “Approximately” 25 mL fermentation was used in each pot fixed in the bottom of the trap and two 11 cm filter papers were placed on upper layer of bait fermentation to provide a suitable helipad for sucking of the adult insects. Each trap was 34 cm long, 20 cm wide, round shaped in which 20 cm funnel/cone-shaped body sieve was attached having 4 cm hole for trapping the insects (Fig. 1).

### Data collection

The sampling was carried out from last week of August to last week of September. Each trap having various insects’ populations as taken into the laboratory, preserved at −36 °C for killing these insects for 2 h. These insects were stored for subsequent laboratory processing comprising identification, drying, spreading, pinning, photographing and labeling. Difficult insect’s specimens were identified with the consultation of the Entomological Department of Shenyang Agricultural University, Shenyang.

The image depicted in Fig. 1 along with graphical abstract are my own data.

The attracted insects sucked the sap from the bait fermentation, moved in upward direction and trapped through hole.

### Calculation of insect diversity

All the trapped insects were separated family-wise, counted separately and calculated diversity of insects such as richness, abundance and evenness compared to control. Insects diversity measured by Shannon–Wiener’s diversity index, Simpson’s index, Margalef’s index and Shannon’s equitability index [32–36].

### Sample extraction from bait material

The bait fermentation was well dried at room temperature (27 °C) and ground gently with pestle and mortar keeping in view the particle size up to 0.1 micron. Methanol and distilled water were used at 4 mL g^−1^ of bait sample; kept for 3 days at room temperature for completion of solvent extraction by maceration method. The waste of bait produced after first extraction reused according to the above procedure and get second extraction and repeated this process for getting third extraction. Each solvent extracts were filtered and dried at room temperature to remove the solvents from the eluents. The first, second and third time extracted dried samples were weighed separately, mixed together, and stored at 4 °C in airtight glass bottles for further use [37]. Physical properties of each extract (color, stickiness and appearance) were recorded visually (Table 1).

**Table 1:** Physical properties of bait fermentation

Solvents extracts	Treatment	Physical properties			
		Color	Opaqueness	Stickiness	Appearance
Methanol	Sugar fermented bait	Dark brown	Shiny	Hard stone like	Immotile
Distilled water		Less dark brown	Dull/crystalline	Sticky	Motile

### Sample preparation for GC–MS screening

Approximately 10 mg dried sample collected from each solvent extract was accurately weighed and put in the centrifuge tube, in which 1 mL of HPLC grade methanol was added to dissolve the sample and vortexed for 2–3 min. About 0.2 g Graphitized carbon black (GCB) was added into the solution and vortexed for 1 min to remove the pigmentations and sterols. If pigmented solution is dark additional 1 mL methanol may be added according to the situation to faint the color of the solution [38]. The mixture was centrifuged for 5 min at 5000 revolutions at 27 °C and repeated two times to obtain good results. The transparent supernatant layer of solvent was detected, collected by micro pipette and stored in glass bottles evaporated to dryness in fume hood. About 1 mL methanol dissolved into the dried samples and stored at − 4 °C for further analysis [38].

### Gas Chromatography–Mass Spectrometry (GC–MS) Analysis

GC–MS investigation was done on (Agilent 6890-5973 N USA) gas chromatograph set with a HP1 slender section (model number TG-5MS) on (30 m × 250 µm × 0.25 µm) polydimethylsiloxane having interfaced (Hewlett Packard 5973 N) mass. The underlying temperature was maintained at 70 °C for 2 min and then increased to 200 °C at rate of 10 °C min^−1^; inlet temperature was set to 250 °C with split ratio of 10:1. MS quadruple pipe and warm aux temperatures were 150 °C and 285 °C, respectively. The MS examine was 35–520 units and helium gas utilized as transporter with 1.0 mL min^−1^ stream rate. The relative yield of mixes crude information was determined depend on gas chromatography (GC) zones with a FID redress factor which is explicit, direct, delicate, exact and precise [37, 39] strategy for estimation.

### Statistical analysis

The week-wise insect diversity collected and three times extracted bait yields analyzed statistically by one-way analysis of variance with Duncan’s Multiple Range test keeping in view* P* > 0.05. All the analysis for recorded data performed by SPSS statistical software (version 13.0; Inc., Chicago, IL, USA).

*Co*-*efficient of determination* (*R*^*2*^) carried out for the model comparison between means of first, second and third times extracted yields by polar solvents compared to root mean square error (RMSE). RMSE gave magnitude of the characteristic variation between predicted and observed data [40] resulted to assess the precision of the model [41].

## Results

**Fig. 2:**
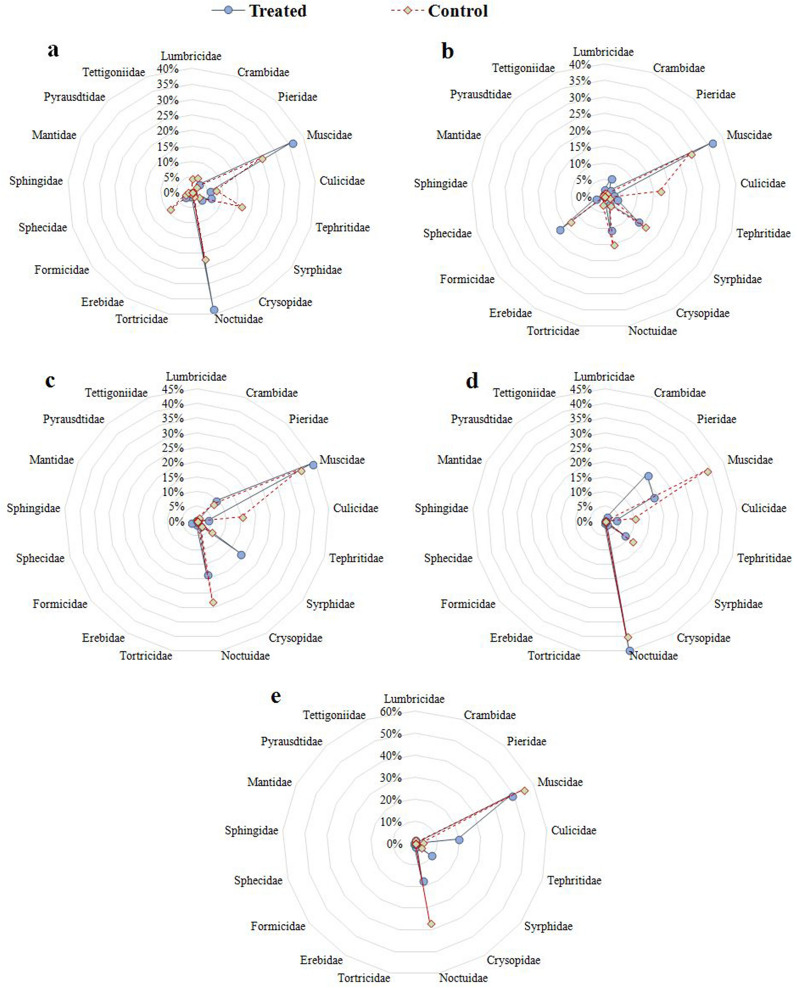
Rank abundance (%) of different insects families collected by bait traps in treated and control. Whereas (**a**) Fourth week of August; (**b**) First, (**c**) Second, (**d**) Third and (**e**) Fourth week of September respectively. These webs depended upon the collected values of insect populations (%) at family level.

The study showed significant (*P* < 0.05) difference in last week of August, 2019 in treated (560 insects related to 11 families) and control traps (219 insects with 13 different families) (Fig. 2a). Insects (248 insects with 13 families) were recorded in treated traps were 24.70% more abundance than in control trap (189 insects with 13 families) during 1st week of September (Fig. 2b). Insects recorded in treated traps (567 different adult insects and 13 different families) were 68.43% more than in control in 2nd week of September (Fig. 2c). Insects in treated traps (315 number of different insects with 8 families) were 77.78% more than in control in 3rd week (Fig. 2d). Insects collected in treated traps (133 different types of insects with 8 families) were 54.89% more than in control recorded in last week of September (Fig. 2e). The insects were identified under microscope according to morph metric characteristics.

The calculated values of Shannon- Wiener’s diversity index during 4th week of August recorded high (1.628) for Noctuidae family followed by Muscidae (1.437) in treated traps. Soybean pests yielded 0.368 in Tephritidae followed by Noctuidae (0.352). The results showed that insects of Lapidoptera, Diptera, Hymenoptera, Neuroptera attracted by the pharomonic activity of bait fermentation were well distributed both in control and treated traps. Maximum rank abundance and diversity of these pests were recorded in treated cage compared to control. Simpson’s diversity index (1-D) ranged from 0.85 (Noctuidae) to 0.86 recorded maximum diversity in treated trap, while rest of the families recorded low or no diversity. Similarly, Muscidae (0.94), Noctuidae (0.95) and Tephritidae (0.97) were more diversified in control compared to rest of the treatments (Table 2(a)).

**Table 2:** Rank of diversity indices of insect orders and different families collected from treated and control traps, (a) Forth week of August, (b) First week of September, (c) Second week of September, (d) Third week of September, (e) Forth week of September

R	Order	Insect families	Diversity indices					
			Treated			Control		
			Hs	SID	SE	Hs	SID	SE
(a) 4th week								
1	Lepidoptera	Noctuidae	1.628	0.85	1.48	0.352	0.95	0.32
2		Crambidae	0	1	0	0.243	0.99	0.22
3		Pieridae	0.316	0.99	0.29	0.15	0.99	0.22
4		Pyrausdtidae	0	1	0	0	1	0
5		Tortricidae	0	0.99	0	0	1	0
6		Erebidae	0.202	0.999	0.18	0.046	1	0
7		Sphingidae	0.046	1	0	0.105	0.99	0.15
8	Diptera	Muscidae	1.437	0.86	1.31	0.329	0.94	0.3
9		Culicidae	0.365	0.99	0.33	0.301	0.99	0.27
10		Tephritidae	0.368	0.99	0.33	0.368	0.97	0.33
11		Syrphidae	0.328	0.99	0.3	0.169	0.99	0.24
12	Hymenoptera	Formicidae	0.275	0.99	0.25	0.316	0.99	0.29
13		Sphecidae	0.105	0.99	0	0.15	0.99	0.22
14	Neuroptera	Crysopidae	0.129	0.99	0.19	0.105	0.99	0.1
15		Lumbricidae	0	1	0	0.23	0.99	0.21
16	Mantodea	Mantidae	0	1	0	0	1	0
17	Orthoptera	Tettigoniidae	0	1	0	0	1	0
Species richness		Ni	5.2	0.71	4.66	2.864	0.85	2.87
		N	9.67			N	10.67	
		R	0.71	Ev		R	1.24	Ev
		d	9.48	0.41		d	10.43	1.21

(b) 1st week								
1	Lepidoptera	Noctuidae	0.35	0.99	0.32	0.356	0.98	0.32
2		Crambidae	0.275	0.99	0.25	0.078	0.99	0.11
3		Pieridae	0.169	0.99	0.15	0.078	0.99	0.11
4		Pyrausdtidae	0	1	0	0	1	0
5		Tortricidae	0	1	0	0	0.99	0
6		Erebidae	0	1	0	0	1	0
7		Sphingidae	0.046	1	0	0	1	0
8	Diptera	Muscidae	0.095	0.87	0.09	0.329	0.92	0.3
9		Culicidae	0.186	0.99	0.17	0.365	0.97	0.33
10		Tephritidae	0.23	0.99	0.21	0.105	0.99	0
11		Syrphidae	0.365	0.98	0.33	0.359	0.97	0.33
12	Hymenoptera	Formicidae	0.359	0.96	0.33	0.343	0.98	0.31
13		Sphecidae	0.186	0.99	0	0	1	0
14	Neuroptera	Crysopidae	0.186	0.99	0.269	0.17	0.99	0.15
15		Lumbricidae	0.15	0.99	0.216	0.08	0.99	0.11
16	Mantodea	Mantidae	0	1	0	0	1	0
17	Orthoptera	Tettigoniidae	0	1	0	0	1	0
Species richness		Ni	2.598	0.81	2.5	2.26	0.83	2.08
		N	11			N	8.33	
		R	1.2		Ev	R	1.05	Ev
		d	10.77		1.084	d	8.09	1.07
(c) 2nd week								
1	Lepidoptera	Noctuidae	0.0408	0.97	0.04	0.347	0.92	0.32
2		Crambidae	0	1	0	0.078	0.99	0
3		Pieridae	0.336	0.99	0.31	0.275	0.99	0.25
4		Pyrausdtidae	0	0.99	0	0	1	0
5		Tortricidae	0	0.99	0	0	1	0
6		Erebidae	0.078	0.99	0.07	0	1	0
7		Sphingidae	0	1	0	0	1	0
8	Diptera	Muscidae	2.2525	0.81	2.05	0.25	0.84	0.23
9		Culicidae	0.328	0.99	0.3	0.354	0.98	0.32
10		Tephritidae	0	1	0	0	1	0
11		Syrphidae	0.0512	0.96	0.05	0.243	0.99	0.35
12	Hymenoptera	Formicidae	0	1	0	0	1	0
13		Sphecidae	0.243	0.99	0.22	0	1	0
14	Neuroptera	Crysopidae	0.243	0.99	0.35	0.129	0.99	0.12
15		Lumbricidae	0.078	0.99	0	0.046	1	0
16	Mantodea	Mantidae	0	1	0	0	1	0
17	Orthoptera	Tettigoniidae	0	1	0	0	1	0
Species richness		Ni	1.0382	0.73	1.77	1.721	0.78	3.17
		N	8.67			N	6.33	
		R	0.63	Ev		R	0.82	Ev
		d	8.48	0.4818		d	6.09	0.93
(d) 3rd week								
1	Lepidoptera	Noctuidae	0.4845	0.80	0.44	0.356	0.84	0.32
2		Crambidae	0.15	0.99	0.14	0	1	0
3		Pieridae	0.268	0.95	0.24	0	1	0
4		Pyrausdtidae	0	1	0	0	1	0
5		Tortricidae	0.078	0.99	0.11	0	1	0
6		Erebidae	0	1	0	0	1	0
7		Sphingidae	0	1	0	0	1	0
8	Diptera	Muscidae	0.316	0.96	0.29	0.354	0.85	0.32
9		Culicidae	0.254	0.99	0.23	0.186	0.99	0
10		Tephritidae	0	1	0	0	1	0
11		Syrphidae	0.35	0.99	0.32	0.202	0.99	0.29
12	Hymenoptera	Formicidae	0	1	0	0	1	0
13		Sphecidae	0	1	0	0	1	0
14	Neuroptera	Crysopidae	0.129	0.99	0.19	0	1	0
15		Lumbricidae	0	1	0	0	1	0
16	Mantodea	Mantidae	0	1	0	0	1	0
17	Orthoptera	Tettigoniidae	0	1	0	0	1	0
Species richness		Ni	1.061	0.71	2.15	1.098	0.68	1.87
		N	7.333			N	3	
		R	0.72	Ev		R	0.63	Ev
		d	7.12	0.5321		d	2.68	1
(e) 4th week								
1	Lepidoptera	Noctuidae	0.338	0.97	0.31	0.333	0.87	0.3
2		Crambidae	0	1	0	0	1	0
3		Pieridae	0	1	0	0	1	0
4		Pyrausdtidae	0	1	0	0	1	0
5		Tortricidae	0.078	0.99	0.11	0	1	0
6		Erebidae	0	1	0	0	1	0
7		Sphingidae	0.046	1	0	0	1	0
8	Diptera	Muscidae	0.28	0.76	0.25	0.366	0.7	0.33
9		Culicidae	0.35	0.96	0.32	0.078	0.99	0
10		Tephritidae	0	1	0	0	1	0
11		Syrphidae	0.254	0.99	0.23	0.078	0.99	0
12	Hymenoptera	Formicidae	0		0	0	1	0
13		Sphecidae	0	1	0	0	1	0
14	Neuroptera	Crysopidae	0.078	0.99	0	0	1	0
15		Lumbricidae	0.078	0.99	0.11	0.046	1	0
16	Mantodea	Mantidae	0	1	0	0	1	0
17	Orthoptera	Tettigoniidae	0	1	0	0	1	0
Species richness		Ni	1.503	0.69	1.34	0.901	0.57	0.63
		N	6			N	3	
		R	0.9	Ev		R	0.68	Ev
		d	5.73	0.84		d	2.66	0.82

*R* Rank, *Hs* Shannon-Weiner index, *SID* Simpson Index of Diversity, *SE* Species Equitability, *Ni* number of individuals, *N* number of families, *R* Menhenick index, *Ev* evenness, *d* Margalef’s Index

Margalef’s index in treated trap was maximum (10.77) followed by control trap (8.09) in 1st week (Table 2(b)) followed by 8.48 and 6.09 in 2nd week (Table 2(c)), 7.12 and 2.68 in 3rd week (Table 2(d)) and 5.73 and 2.66 recorded in 4th week of September (Table 2(e)). Our results showed that maximum species richness (Margalef’s index) was recorded in control (10.43) compared to treated (9.48) in 4th week of August (Table 2(a)). The yielded values of this index indicated that insects investigated in treated traps have more richness compared to control. Shannon’s equitability’s calculated that insect populations recorded high in Noctuidae (1.48) followed by Muscidae (1.31) in treated traps (Table 2(a)). It was observed clearly that the insects collected in treated traps recorded high equitability of the Noctuidae and Muscidae families in both treatments.

Similar eveness was recorded in Syrphidae (0.33), Formacidae (0.33) and Noctuidae (0.32) in treated traps (Table 2(b)). Maximum evenness was observed in Muscidae family (2.05) followed by Crysopidae (0.35) and Pieridae (0.31) in treated trap compared to other insects families during 2nd week of September (Table 2(c)). In 3rd week elevated evenness was recorded in Noctuidae (0.44) followed by Syrphidae (0.32) and Muscidae (0.29) in treated cages (Table 2(d)) compared to 4th week of September (Table 2(e)).

Furthermore the economic analysis of bait fermentation proved that it was eco-friendly, consumed only 0.64 RMB/0.09 USD per location, and did not spread toxic chemicals to the environment and surrounding area of human populations.

### Model validations

The dry yield (g) recorded from each solvent extract showed significant (P < 0.05) linear arc curve within treatments during first, second and third time extraction. *Coefficient of determination* recorded positive relationship by bait + methanol (*R*^*2*^ = 0.87 and RMSE = 0.97) extraction and materials extracted from bait + distilled water (*R*^*2*^ = 0.87 and RMSE = 0.93) indicated better performance of the model fitness (Table 3).

**Table 3:** Coefficient of determination (*R*^*2*^) showing the relationship between low and high polarity extraction solvent on yield (g) and root mean square error (RMSE) of bait fermentation

Treatments	Extraction yield (g)			Regression Equation	*R*^*2*^	*RMSE*
	First	Second	Third			
Bait + distilled water	0.9579a	0.2208b	0.0705c	− 0.4437x + 1.3038	0.87	0.97
Bait + methanol	0.9071a	0.2417b	0.1115c	− 0.3978x + 1.2157	0.87	0.93

Whereas level of significance was* P* = 0.05, *RMSE* root mean square error, *R*^*2*^ Coefficient of determination

### GC–MS screening bait fermentation

The low polarity solvent (distilled water) was involved for the extraction of volatile compounds from bait fermentation through GC–MS analytical technique. The results of GC–MS showed that twenty-two different compounds were detected at different retention times (RT) with 99.99% correspondence of bioactive compounds. Similarly the high polarity solvent (methanol) was also checked for compounds determination. Twenty-two different bioactive compounds were detected (Table 4).

**Table 4:** Chemical composition of bait fermentation with different solvents by GC–MS

Distilled water extract				Methanol extract				Refs.
RT	Chemical name	M.F.	MM	RT	Chemical name	M.F.	MM	
3.23	*p*-Xylene	C_8_H_10_	106	3.23	*p*-Xylol	C_8_H_10_	106	NC
3.83	*N*-Methyl-β-phenethylamine	C_9_H_13_N	135	3.83	Carboxyacetic acid	C_3_H_4_O_4_	104	[42–44]
3.93	Hemimellitene	C_9_H_12_	120	3.92	Rubeanic acid	C_2_H_4_N_2_S_2_	120	NC, [45]
4.01	Pseudocumo	C_9_H_12_	120	4.00	Nitrosomethylurea	C_2_H_5_N_3_O_2_	103	NC, [46]
4.09	Octamethylcyclotetrasiloxane	C_8_H_24_O_4_Si_4_	296	4.08	Octamethyltetrasiloxane	C_8_H_24_O_4_Si_4_	296	NC
4.15	Methoxyphenamine, *N*-desmethyl	C_10_H_15_NO	165	4.13	Nitrosomethylurea	C_2_H_5_N_3_O_2_	103	[46]
4.32	Trimethylbenzene	C_9_H_12_	120	4.30	1-Aminoglycerol	C_3_H_9_NO_2_	91	NC, [47–51]
4.88	Allylbenzene	C_9_H_10_	118	4.87	Benzocyclopentane	C_9_H_10_	118	NC
5.53	Hendecane	C_11_H_24_	156	5.52	2-Methylpiperazine	C_5_H_12_N_2_	100	[52]
5.60	1,2:7,8-Dibenzocarbazole	C_20_H_13_N	267	5.64	Glyoxylic acid	C_2_H_2_O_3_	74	[53, 54]
5.65	Dexamphetamine	C_9_H_13_N	135	6.40	3,4-Furandiol, tetrahydro-, trans-	C_4_H_8_O_3_	104	[45]
6.42	Dexamphetamine	C_9_H_13_N	135	6.57	Tetralin	C_10_H_12_	132	[45, 55]
6.59	Naphthalene-1,2,3,4-tetrahydride	C_10_H_12_	132	6.95	Camphor tar	C_10_H_8_	128	NC
7.32	Dexamphetamine	C_9_H_13_N	135	7.31	Tetraacetyl-d-xylonic nitrile	C_14_H_17_NO_9_	343	[45]
7.48	Dexamphetamine	C_9_H_13_N	135	7.47	*o*-Methylisourea hydrogen sulfate	C_2_H_8_N_2_O_5_S	172	[45, 56]
9.37	Fluoroacetamide	C_2_H_4_FNO	77	7.99	1,4-Anhydro-l-threitol	C_4_H_8_O_3_	104	[57]
9.53	5-[4-(Dimethylamino)cinnamoyl]acenaphthene	C_23_H_21_NO	327	9.52	l-Cysteine disulfide	C_6_H_12_N_2_O_4_S_2_	240	[47, 58]
11.1	2-Aminoundecane	C_11_H_25_N	171	9.72	Tetraacetyl-d-xylonic nitrile	C_14_H_17_NO_9_	343	[59, 60]
11.5	Propionic acid amide	C_3_H_7_NO	73	11.13	*N*-Propylacetamide	C_5_H_11_NO	101	[61]
13.3	l-Alanine-4-nitroanilide	C_9_H_11_N_3_O_3_	209	11.50	1,3,5-Trioxacycloheptane	C_4_H_8_O_3_	104	[62]
14.3	1,5-Diphenyl-2H-1,2,4-triazoline-3-thione	C_14_H_11_N_3_S	253	13.37	l-Cysteine disulfide	C_6_H_12_N_2_O_4_S_2_	240	[63, 64]
15.8	1,2-Dimethylpropylamine	C_5_H_13_N	87	14.29	1,5-Diphenyl-2H-1,2,4-triazoline-3-thione	C_14_H_11_N_3_S	253	[65, 66]

*RT* Retention time, *M.F* molecular formula, *MM (g/mol)* molar mass gram/mole, *Refs.* references, *NC* non-target compound)

## Discussion

The present investigations recorded diversity indices of seventeen insect’s families with in six orders collected in treated traps suggested significant (P < 0.05) abundance ranged 24.70–77.78%. These results are in agreement with the scientists who reported that the insect populations of Noctuidae, Pieridae, Lycaenidae, Nymphalidae, Hesperiidae families increased by sugar fermented traps [30, 67, 68]. Our results suggested that the height of webs (Fig. 2) depended upon the collected values of insect populations at family level. High value of Shannon-Wiener’s diversity index recorded in Noctuidae followed by Muscidae. These investigations are in accordance to the researchers who described higher Shannon index value (*P* < 0.01) in their experiments [69]. Our investigations suggested significant (P < 0.05) soybean pest yielded high in Tephritidae during last week of August are in accordance to the researchers reported similar recommendations [70]. Margalef’s index recorded maximum in treated trap followed by control in 1st week of September. The insects yielded with Shannon’s equitability’s investigated high value in Noctuidae compared to Muscidae in treated traps. Maximum evenness recorded in Muscidae are in line with the researchers who revealed species uniformity or evenness of cabbage pests [69]. Our results are in agreement with the researchers who reported that fermented bait is successful biocontrol agent to attract the major and minor pests [71–74]. Lepidopterous moth attracted by the pharomonic activity of fruits fermented baits for collecting their protein food and trapped easily [75, 76]. The moths of different families were attracted to baits traps may give reliable estimates of captured moth diversity [77].

Our hypothesis was confirmed that insect diversity of different families is high and first time dry mass yield (g) extracted from different polar solvents recorded significant (P < 0.05) results compared to rest of extractions. According to the literature cited, our traditionally prepared bait fermentation contained bioactive compounds, which attract the respective insects and charged only 0.64 RMB or 0.09 USD per location. The bait fermentation is cheap, economical and easy to install source for the attraction of insects in current scenario. The different polarity solvents of bait fermentation were analyzed through GC–MS analytical technique and showed that twenty-two different types of bioactive compounds were identified in both cases. Other researchers also reported that fermented bait could be used as attractant for Noctuidae insects [78–81]. Male and female flies feed on nectar and organic matter, so they are commonly attracted to waste receptacles and other forms of organic matter [82]. The researchers reported that metabolites release volatile fumes into the environment that convey specific message helpful for the attraction of different kinds of insects [34]. The researchers showed that fruit baits are necessary items in food ingredients for the attraction of tephritidae [83–85]. Many insects of order Diptera, Lapidoptera, Hymenoptera and Neuroptera are attracted towards protein foods in bait trap, which are in line with the researchers who also reported that insects are attracted through chemicals signaling of organic compounds [31, 86]. This bait fermentation is cheap, non-toxic, safe and environment friendly due to their natural origin. In our study, we utilized typical beer, which gave satisfactory results according to the scientists who reported that lighter beer also attract the insects tremendously [87, 88]. This bait is cost-effective, economical, safe used for Integrated Pest Management (IPM) [77].

## Conclusions

Insects belonging to Lapidoptera, Diptera and other orders are attracted by the pharomones activity of bait fermentation, which undicates that major and minor pests and domestic insects (mosquito, house flies) are easily trapped. The bait treated trap captured the maximum abundance of insects populations compared to control and yielded higher diversity values. The fermented volatile organic compounds in bait attracted the insects. Both male and female insects were attracted successfully in bait traps, which play a vital role in Integrated Pest Management (IPM). Entomologists, ecologists and researchers are advised to innovate bait formulations for the use of broad spectrum field experiments and incearse the trapping efficiency of the insects. Additional investigations would be conducted on the chemical ecology of the target insect-pests and bait fermentation along with their interaction mechanism through olfactory responses of insects in future.

## Data Availability

All the data of the study are included in this manuscript; hence there are no additional data with the authors.

## Abbreviations

GC–MS: Gas Chromatography and Mass Spectrometry
R^2^: Co-efficient of determination
RMB: Renminbi
USD: United States Dollar
^o^N: Degree North
^o^E: Degree East
pH: Power of hydrogen ion
HPLC: High Performance Liquid Chromatography
GCB: Graphitized carbon black
µm: Micrometer
FID: Flame ionization detector
R: Rank
Hs: Shannon-Weiner index
SID: Simpson index of diversity
SE: Species equitability
Ni: Number of individuals
N: Number of families
R: Menhenick index
Ev: Evenness
d: Margalef’s Index
RMSE: Root mean square error
RT: Retention times
Refs: References
IPM: Integrated pest management
